# Ru-Catalyzed Polyethylene Hydrogenolysis under Quasi-Supercritical Conditions

**DOI:** 10.1021/jacsau.5c00006

**Published:** 2025-04-16

**Authors:** Sungmin Kim, Boda Yang, Oliver Y. Gutiérrez, Wei Zhang, Carlos Lizandara-Pueyo, Piyush Ingale, Ivana Jevtovikj, Reni Grauke, Janos Szanyi, Huamin Wang, Stephan A. Schunk, Johannes A. Lercher

**Affiliations:** † Institute for Integrated Catalysis and Physical Science Division, Pacific Northwest National Laboratory, Richland, Washington 99354, United States; ‡ BASF SE, Carl-Bosch-Straße 38, 67056 Ludwigshafen am Rhein, Germany; § hte GmbH, Kurpfalzring 104, 69123 Heidelberg, Germany; ∥ Institut für Technische Chemie, Universität Leipzig, Linnéstraße 3, 04103 Leipzig, Germany; ⊥ Department of Chemistry and Catalysis Research Institute, TU München, Lichtenbergstrasse 4, 85748 Garching, Germany

**Keywords:** catalytic polyolefin conversion, hydrogenolysis, solvent, quasi-supercritical conditions, kinetic isotope effect in hydrogenolysis

## Abstract

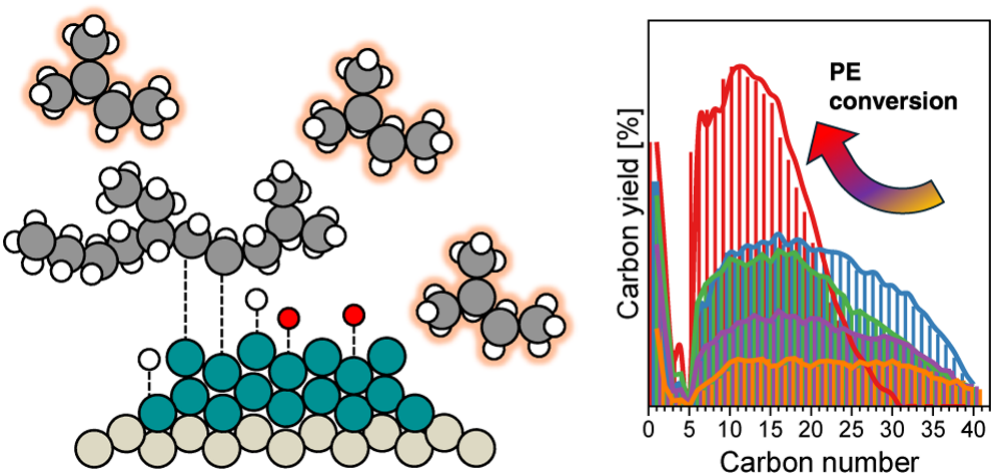

Ru/C-catalyzed polyethylene (PE) and hydrocarbon hydrogenolysis under quasi-supercritical fluid of isopentane was kinetically and mechanistically investigated. PE hydrogenolysis with C–C and C–H cleavage showed zeroth order, suggesting strong adsorption of hydrocarbons. PE yielded broad product distribution of heavy (C_21–40_) and diesel-range (C_11–20_) hydrocarbons in the primary step of hydrogenolysis due to stochastic C–C cleavage over Ru surface. Catalytic hydrogenolysis of *n*-hexadecane, squalane, and light hydrocarbons such as *n*-pentane, iso-pentane, and *n*-hexane further described C–C cleavage reactivity between primary and secondary carbons, i.e., ^1^C–^2^C and ^2^C–^2^C, which has an order of magnitude higher hydrogenolysis rate than that involving a tertiary carbon. The PE saturated Ru surface and lower C–C cleavage reactivity of tertiary carbon in iso-pentane, therefore, imited sovlent conversion during hydrogenolysis, whereas leading to selective PE conversion. Using hexadecane, we observed comparable hydrogenolysis rates between H_2_ and D_2_ (*k*_H_*/k*_D_ ∼ 1), indicating the kinetically relevant step of C–C cleavage with facilitating C–H cleavage and rehydrogenation. However, the normal kinetic isotope effect between hexadecane and deuterated hexadecane (*k*_C_16_H_34__*/k*_C_16_D_34__ ∼ 5) revealed that the dehydrogenation, i.e., C–H cleavage, can be kinetically involved in the hydrogenolysis kinetic. By considering the 8-fold lower H-D exchange rate with deuterated hexadecane compared to n-hexadecane, the lower rate for hydrogenolysis and H-D exchange with deuterated hexadecane can be attributed to the C–D bond dissociation energy being 3 kJ/mol higher than that of the C–H bond. Increasing H_2_ pressure favors internal C–C bond cleavage over terminal one. This minimizes the formation of lower hydrocarbons, particularly methane. However, the increase in H_2_ pressure increases the coverage of adsorbed hydrogen on the Ru particles due to competitive adsorption of H_2_ and polyethylene, which, in turn, reduces the polyethylene conversion rates.

## 1 Introduction

The global production of plastics grows steadily and is projected to reach 445 million metric tons in 2025,^1,2^ dominated by polyethylene, polypropylene, polyvinyl chloride, polyethylene terephthalate, and polystyrene.^3−5^ Polyolefins, i.e., most ofpolyethylene, and polypropylene, significantly contribute to approximately 66% of plastic waste.^6^ The characteric feature of polymers with versatility, durability, and cost-effectiveness has led to an impressive growth rate of industrial production.^6,7^ Moreover, the high volume of polymer consumption has further led to pollution due to the absence of rigorous waste management.^8−10^ As these polymers constitute approximately 5% of the total carbon processed, their waste, however, can be considered as an untapped reservoir of carbon. Thus, recycling or upcycling polymer waste is crucial to mitigate the deterioration of the living environment.

Mechanical recycling is currently the prevalent approach.^11,12^ However, thermal treatment in the presence of additives and contaminants during polymer recycling^13^ typically lead to the degradation of both the purity and physical properties of recycled plastic over time.^14−16^ While incineration of plastics recovers the energy equivalents in the material, chemical recycling may offer a more sustainable and atom-efficient solution, assuming that the resulting recyclates can be integrated into current product streams.^16,17^

Chemical recycling enables depolymerization of the macromolecules into smaller entities through catalyzed cleavage of the polymer chemical bonds. However, the challenge lies in the highly stable C(sp^3^)–C(sp^3^) and C(sp^3^)–H bonds of polyolefins, which leads to high endothermicity of C–C and C–H cleavage in chemical polyolefin deconstruction.^18,19^ These are more stable than the C-heteroatom bonds of functionalized polymers, such as terephthalate, polyesters, and polyamides.^20−22^ In order to allow high conversion levels at lower temperatures, the endothermic polyolefin depolymerization has to be coupled, for example, with exothermic hydrogen addition through hydrogenolysis,^23−29^ hydrocracking,^30−32^ or C–C bond formation via alkylation,^33^ and alkane metathesis.^34^

Catalytic hydrogenolysis has been investigated as one of the most promising approaches to depolymerize polyolefins operating at relatively mild temperatures (473–573 K) and affording tunable product distribution. In recent studies, Pt-,^35−38^ Ru-,^24−26,28,38,39^ Rh-,^24,38^ Pd-,^24,38^ Ir-,^24,38^ Co-,^38,40,41^ and Ni-based^42^ catalysts^38,43,44^ have been used for polyolefin hydrogenolysis. Several investigations on supported Ru catalysts, i.e., Ru/C,^24−27^ Ru/zeolite,^45,46^ Ru/CeO_2_,^28^ Ru/TiO_2_,^23,47^ and Ru/WZrO_*x*_,^48^ as well as Ru single site^49,50^ or single atom^28,51,52^ have suggested that it may be the most suitable metal-based catalyst for hydrogenolysis.^44^ The design of such catalysts requires to elucidate the link between geometric and electronic properties and their activity and selectivity.^28,46,53^ Defining the kinetics and mechanisms of polyolefin hydrogenolysis is, however, required to elucidate a holistic understanding of the mechanism and to serve as a guide for the insightful instruction for new and alternative catalyst candidates or reaction regimes.

Currently, most polyolefin hydrogenolysis studies have been conducted in the absence of a solvent using reaction conditions only suitable for polyolefin melts with rapidly changing reaction conditions as the conversion progresses.^23−26,28,36,38,42^ This expectedly leads to ill-defined mass and heat transfer as density, viscosity, and molecular structure lead to polymer feedstock changes, significantly impacting overall catalytic performance. Conformation of polyolefins in solvents (studied in combination with molecular dynamic calculations) showed that alkanes such as *n*-hexane induce a coiled polymer structure on the catalyst surface and higher reactivity than in melts.^27^ Conceptually, the impact of the supercritical state should even be more positive, improving polyolefin solubility as well as faster mass transport.

Thus, we investigated Ru/C-catalyzed polyethylene hydrogenolysis in the presence of hydrocarbon solvents under supercritical conditions. Preliminary measurements showed that Ru is highly active for polyethylene hydrogenolysis (Figure S1), where a comparable conversion rate with different amounts of catalyst (Figure S1d) allows us to disregard the mass transfer limitation, which significantly influences hydrogenolysis reactivity by a factor of 4.^54^ The depolymerization under supercritical conditions, which ensures high mobility of the substrate into the catalyst, leads to selective hydrogenolysis of polyethylene, minimizing methane formation and negligible solvent conversion. The consecutive C–C cleavage of the polyolefins upon primary and secondary hydrogenolysis reactions gradually changes the selectivity from heavy oils (C_20–40_) to gasoline-ranged hydrocarbon (C_6–10_) with a clear preference for linear over branched products. Ru-catalyzed hydrogenolysis of model compounds of hexadecane and squalane in this work shows that cleavage of C–C bonds between primary and secondary carbons, i.e., ^2^C–^1^C or ^2^C–^2^C compared to tertiary ^3^C–*^x^*C, is controlling hydrogenolysis reactivity as well as product selectivity.

## 2 Results and Discussion

### 2.1 Selecting the Solvent and Reaction Conditions for Polyethylene Conversion

**Figure 1:**
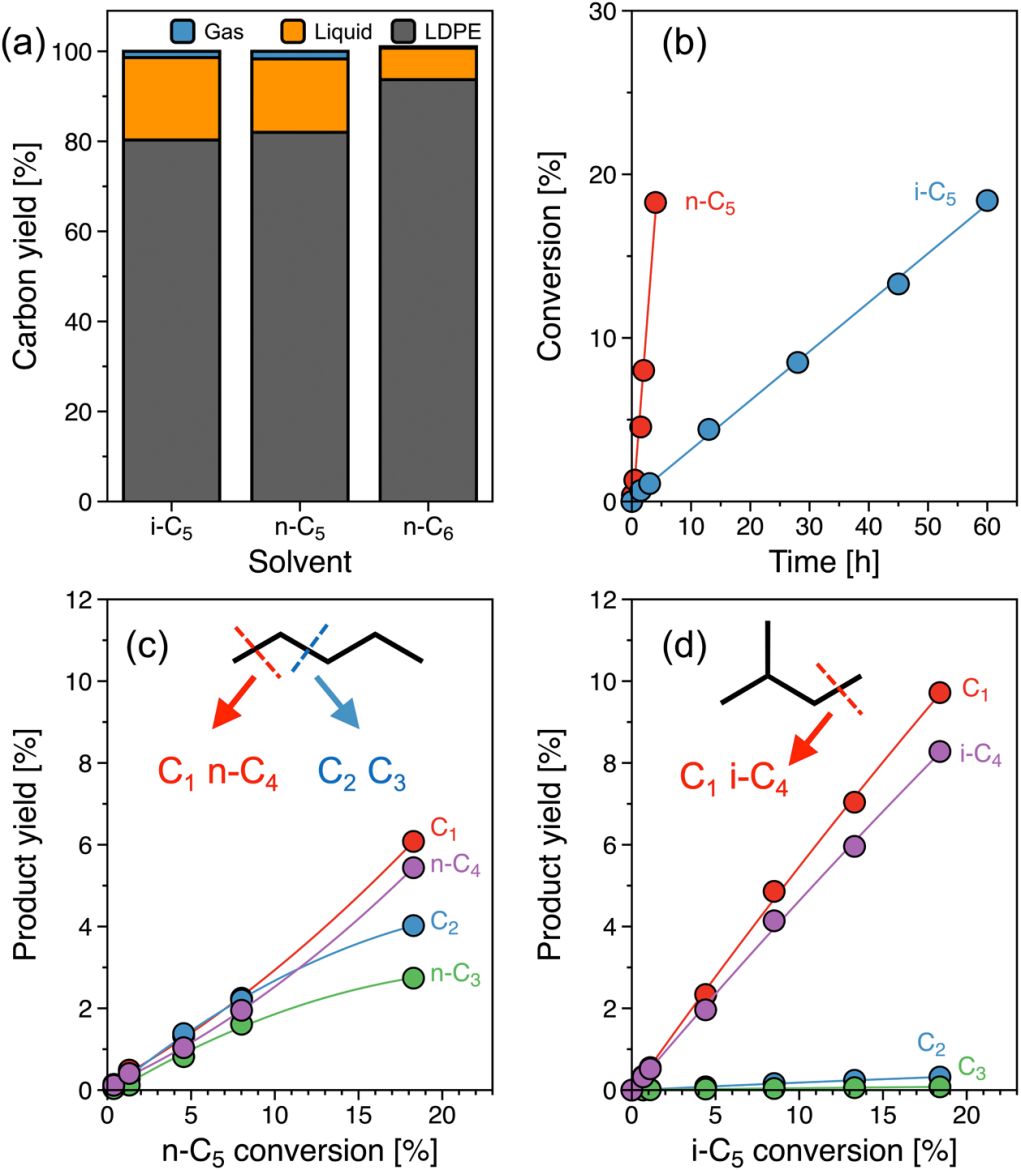
(a) Carbon yield in gas (C_1–4_), liquid (C_5–40_), and solid residue after Ru/C-catalyzed PE hydrogenolysis with different solvents of *n*-C_5_, *i*-C_5_, and *n*-C_6_. The carbon balance is ∼100%. The reaction conditions: *T* = 483 K, *P*_H_2__ = 30 bar, 1 g of PE, 30 mg of catalyst, 40 mL of solvent, and 2 h of reaction. (b) Ru/C-catalyzed *n*-C_5_ and *i*-C_5_ conversion as a function of reaction time and mol-based product yield with the (c) *n*-C_5_ and (d) *i*-C_5_ conversion. Reaction conditions: *T* = 483 K, *P*_H_2__ = 30 bar, 40 mL of solvent, 150 mg of catalyst, and 0.1–60 h of reaction. The insets in (c, d) represent ^3^C–^2^C (red) and ^2^C–^2^C (blue) cleavage of the solvents.

The physicochemical and thermodynamic properties of the hydrocarbon solvent can critically impact polyethylene (PE) hydrogenolysis. With hydrocarbons being formed during hydrogenolysis, we first investigated several lighter hydrocarbons as potential solvents, i.e., *n*-pentane (*n*-C_5_), iso-pentane (*i*-C_5_), and *n*-hexane (*n*-C_6_). The reaction temperature of 483 K was chosen because it was close to but clearly above the critical temperature of the pure solvents *n*-C_5_ (469 ± 1 K) and *i*-C_5_ (464 ± 5 K). We surmise that PE is effectively solvated under these conditions, facilitating PE interaction with the Ru surface. Note that the phase envelope predicted by the Soave–Redlich–Kwong equation of state estimated the critical temperature to decrease by 1 K, i.e., 468 and 463 K for *n*-C_5_ and *i*-C_5_, respectively, when mixed with *n*-hexadecane (Figure S2), which we regard as a model compound of long-chain hydrocarbons. The rates of PE conversion in *n*-C_5_ and *i*-C_5_ were similar (18 and 20% conversion after 2 h at 483 K), while the rate was 6-fold lower in *n*-C_6_ (3% of PE conversion, Figure 1a). PE conversion reached 21% at the critical temperature of *n*-C_6_ and n-hexadecane mixture (518 K)for 2 h of rection time (Figure S3). This can be due to increased reaction temperature as well as the positive impact of the supercritical environment.^54^

To better understand the reactivity of potential solvents, we investigated the reactivity of Ru/C-catalyzed hydrogenolysis of *n*-C_5_ and iC_5_. *n*-C_5_ and *i*-C_5_ conversions after respective 4 and 60 h of reaction time at 483 K reached approximately 18%, which implies normalized solvent consumption rates of 2.4 and 0.15 mol_solvent_/mol_Ru_/s. The observation that the hydrogenolysis rate of *n*-C_5_ is an order of magnitude higher than that of *i*-C_5_ aligns well with the understanding that the hydrogenolysis rate decreases with an increasing number of substituted carbon atoms in the C–C bond.^55,56^ This is mostly attributed to the higher free-energy barrier at the transition state and steric hindrance induced by the methyl group for C–C bond cleavage of a tertiary carbon.^55^ In this regard, iso-butane (*i*-C_4_) can be an ideal solvent due to its minimal reactivity, enabling efficient solvent recycling throughout the hydrogenolysis process. Furthermore, it can be linked to the lower hydrogenolysis reactivity of polypropylene than polyethylene, probably due to the steric hindrance over Ru.^23^

The facile C–C cleavage also influences product selectivity during solvent hydrogenolysis. Ru/C catalyzed *n*-C_5_ into linear C_1–4_ hydrocarbons upon hydrogenolysis (Figure 1c). The equivalent molar selectivity of C_1_ (30%) and *n*-C_4_ (29%), as well as C_2_ (21%) and *n*-C_3_ (20%), at 5–10% of *n*-C_5_ conversion, describes primary terminal (^2^C–^1^C) and central (^2^C–^2^C) C–C cleavages, respectively. The comparable central and terminal C–C cleavage indicates a stochastic C–C cleavage on the Ru surface. With increased *n*-C_5_ conversion, we observed that the C_1_ and *n*-C_4_ formation increased to 33 and 31%, but C_2_ (21%) and *n*-C_3_ (15%) decreased. This indicates secondary terminal C–C cleavage of *n*-C_2–4_, which enhances C_1_ formation. Those findings show good agreement with transition metal-catalyzed, i.e., Ni^57^ and Rh,^58^*n*-C_5_ hydrogenolysis. On the other hand, for *i*-C_5_, selectivity to C_1_ and *i*-C_4_ was predominantly observed (Figure 1d), attributed to the lower rates with bonds involving tertiary carbon atoms. The findings suggest that the C–C cleavage rates on Ru/C follow the order ^2^C–^1^C ≥ ^2^C–^2^C ≫ ^3^C–^1^C or ^3^C–^2^C. It should be emphasized, however, that the Ru/C-catalyzed hydrogenolysis of *n*-C_5_ and *i*-C_5_ becomes negligible in the presence of PE or *n*-hexadecane. This is attributed to the stronger adsorption of the heavier hydrocarbons on the Ru particles.^59^

### 2.2 General Conversion Path for PE Hydrogenolysis

**Figure 2:**
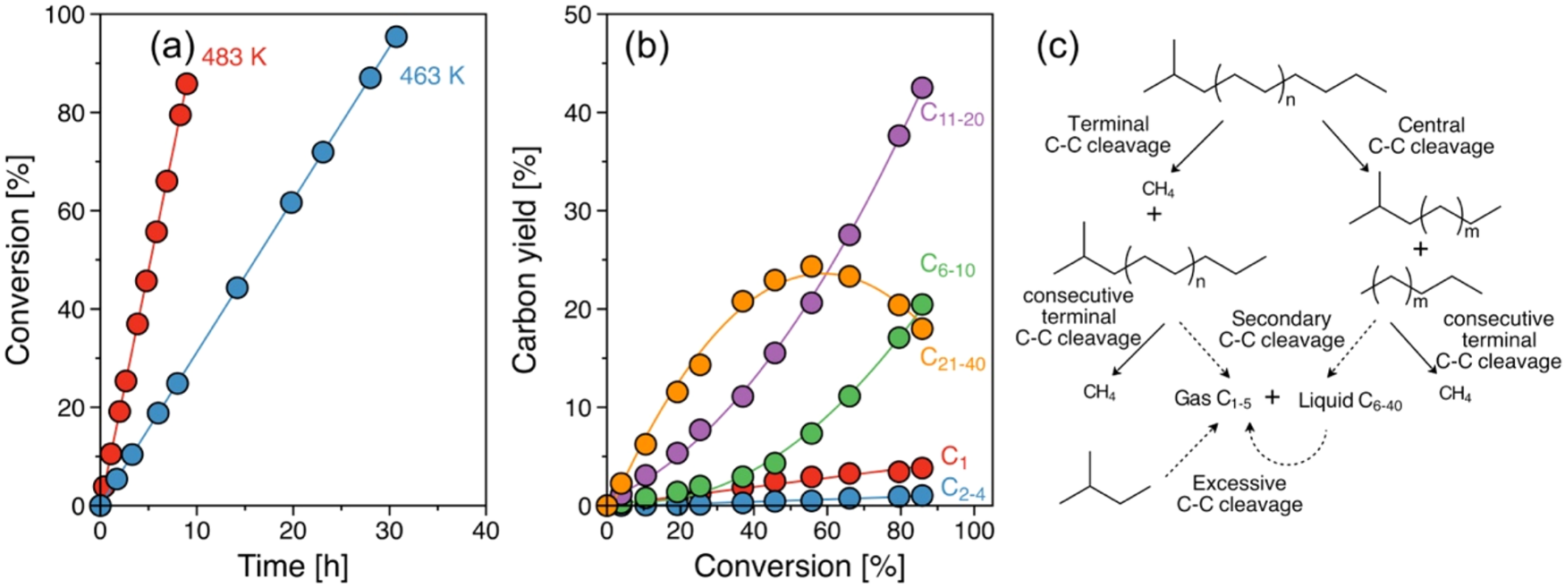
(a) PE conversion with reaction time. (b) Carbon yield of gas (C_1_ and C_2–4_) and liquid (gasoline C_6–10_, diesel C_11–20_, and heavy oil C_21–40_) products as a function of PE conversion at 483 K. Reaction conditions: *T* = 463 and 483 K, *P*_H_2__ = 30 bar, 1 g of PE, 25 mg of catalyst, 40 mL of *i*-C_5_, and 0.1–30 h of reaction. (c) Proposed reaction network for the hydrogenolysis of PE over Ru/C in the presence of *i*-C_5_ solvent with terminal, central, and consecutive terminal C–C cleavages.

To gain a better understanding of PE hydrogenolysis on Ru/C, we further investigated the kinetics and product distributions with varying PE conversion. The PE conversion was adjusted by varying the reaction time and the amount of catalyst. We observed the comparable PE conversion rate normalized by surface Ru sites regardless of the amount of catalyst, indicative of inconsiderable mass transfer limitation (Figure S1d). PE conversion linearly increased with reaction time at 443–503 K (Figures 2a and S4). The constant PE consumption rate regardless of the extent of PE hydrogenolysis points to a zero-order kinetics, leading to the conclusion that the Ru surface is saturated with strongly adsorbed PE molecules.^23^ Statistical mechanics and transition state theory for hydrocarbon hydrogenolysis indicate that the rate constants increase with the length of alkanes due to van der Waals interactions with metal surfaces as well as a substantial increase of activation entropy.^41,48,59^ This finding is consistent with the selective PE hydrogenolysis even in the presence of a solvent, which is due to the preferential adsorption of polymers over small alkanes during hydrocracking and hydrogenolysis.^23,24,32^

The carbon yield of gas (C_1–4_) and liquid (C_6–40_) products along with PE conversion are shown in Figures 2b and S5. In these figures, the C_5_ carbon yield is not included, as *i*-C_5_ was used as a solvent. The liquid products are grouped by the carbon numbers C_6–10_, C_11–20_, and C_21–40_, representing gasoline, diesel, and lubricant-range hydrocarbons. Upon PE hydrogenolysis, we observed predominant selectivity to longer-chain hydrocarbons C_21–40_ (∼50–55% at up to 45% of PE conversion). However, the carbon yield of C_21–40_ decreased with increasing PE conversion to 90%, while a considerable increase in C_6–10_ (50%) and C_11–20_ (24%) occurred. The variation in product distribution with PE conversion indicates that Ru/C catalyzes initial PE hydrogenolysis to C_11–20_ and C_21–40_ products, preventing the product readsorption after the primary C–C cleavage. Upon the primary PE hydrogenolysis, the methane yield is higher in the gas products, albeit the limited number of terminal carbon in PE, indicative of the consecutive cleavage of C–C bonds in the PE chain. Nevertheless, higher carbon selectivity for liquid alkanes over methane is attributed to the multiple contact points within the polymer chain for C–C cleavage, resulting in the formation of liquid products.

The primary products subsequently undergo secondary C–C cleavage to C_6–10_ with the further increase of PE conversion. At higher conversions, the formation of lighter hydrocarbons is attributed to the complete conversion of polymer strands, allowing primary products to adsorb. The adsorption of solvent molecules is still restricted under these conditions. However, at reaction times extending 20 h (Figure S5f), the complete PE conversion resulted in a significant increase in gaseous product formation and a carbon yield exceeding 100% relative to the initial amount of PE, indicating solvent conversion.

A simplified reaction network can be derived by combining these observations (Figure 2c). Central and terminal C–C cleavage are the primary routes, dominating the reaction below 50% PE conversion. The products undergo secondary C–C cleavage once a major fraction of the polymer has been converted, then yielding methane and smaller liquid products. As the reaction approaches complete conversion of PE, the primary and secondary products, as well as the solvent, continue to react and increasingly convert into small molecules, including methane.

Next, we conducted studies to explore how reaction parameters affect PE consumption rate and product distribution in the kinetic regime below 20% of PE conversion yielding only primary PE hydrogenolysis. In a series of experiments conducted at varying temperatures and initial amounts of PE (Figure S6a), the conversion rate of PE hardly increased with higher amounts of PE added to the reaction mixture at temperatures between 443 and 503 K. This finding is in line with the observation of the linear correlation between the amount PE converted and reaction time, indicative of zero-order kinetics for PE hydrogenolysis, which further indicates the saturation of PE on the Ru surface. Interestingly, the reaction remained zeroth order with respect to the polymer even under supercritical conditions above 483 K, while the activation energy (gradually) decreased from 127 to 88 kJ/mol (Figure S6b). Because under these reaction conditions, the measured activation energy equals the true activation energy, this suggests that either the binding of adsorbed hydrogen (the degree of dehydrogenation of the alkane at the surface) or the reaction order in H_2_ has changed.

**Figure 3:**
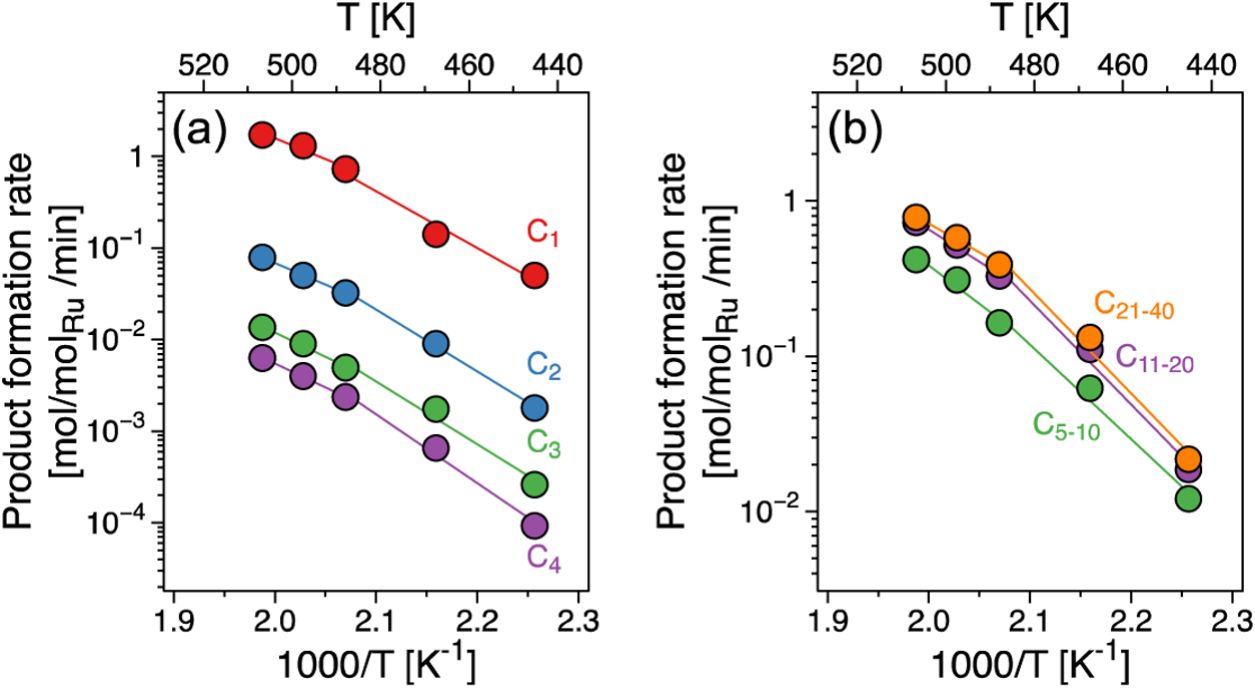
Kinetics of PE hydrogenolysis. formation rate of (a) gaseous and (b) liquid products as a function of reaction temperature Reaction conditions: *T* = 443–503 K, *P*_H_2__ = 30 bar, 1 g of PE, 20–300 mg of catalyst, 40 mL of *i*-C_5_, and 2 h of reaction. The solid lines fit to the Arrhenius equation. The reaction rates are determined at conversions below 20%, ensuring a kinetic regime.

The variations of the product yields with temperature and the corresponding Arrhenius-type plots are compiled in Figure 3a,3b. Identical activation energy changes for the formation of gas and liquid alkanes from 127 ± 9 to 88 ± 10 kJ/mol were observed above 483 K, which shows good agreement with the activation energy changes in PE consumption rate (Figure S6b). This indicates that the selectivity is not affected by temperature at comparable PE conversion (Figure S7). One should note, however, that all temperature dependencies bend at higher than the supercritical temperature of ∼463 K, indicating that the adsorbed state of reactants changes and the rate dependence upon temperature decreases with phase changing.

### 2.3 On the Mechanism of PE and Alkane Hydrogenolysis

**Figure 4:**
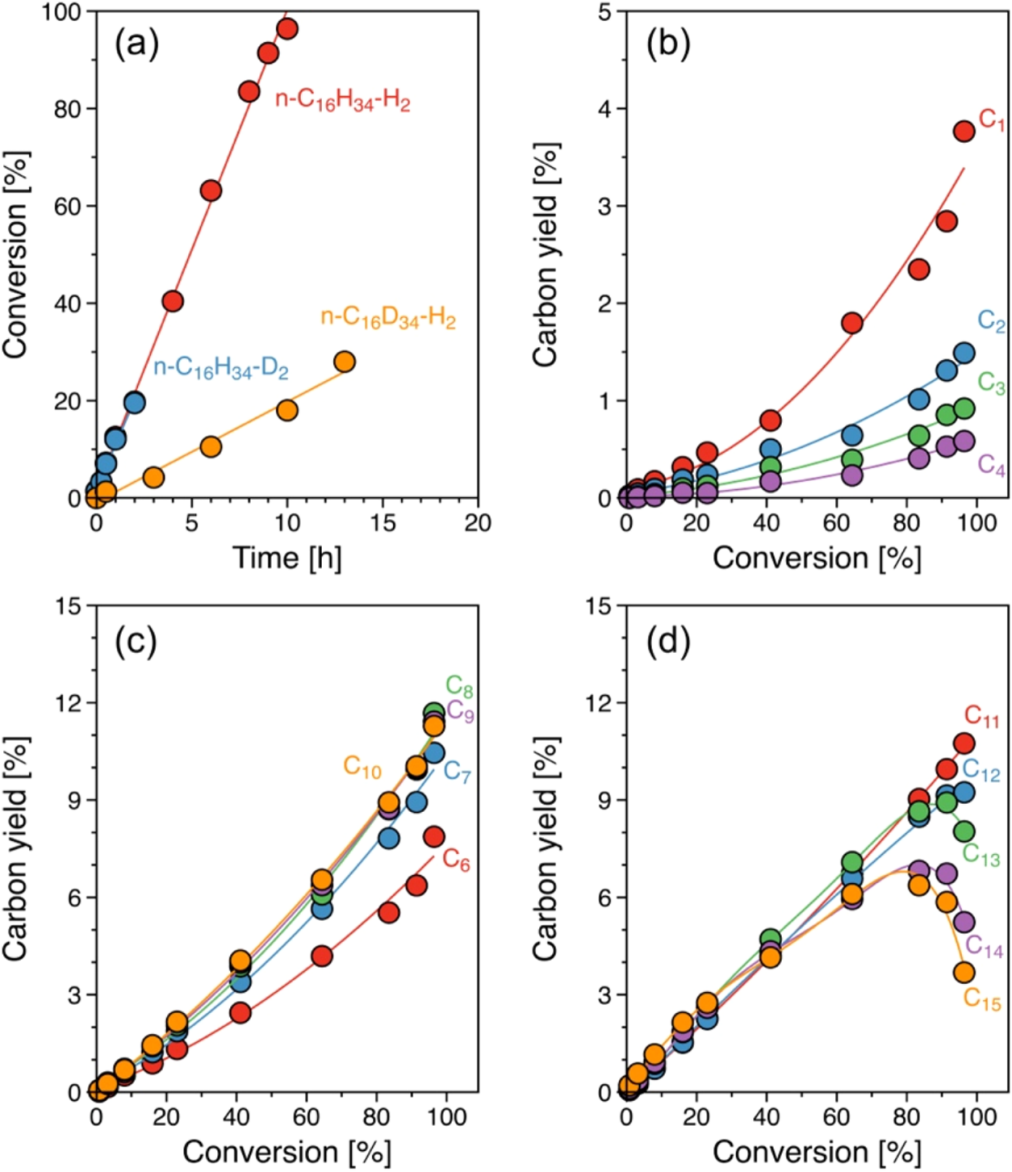
(a) Hexadecane conversion with reaction time. (b) Carbon yield of (b–d) gas (C_1_ and C_2–4_) and liquid products (gasoline C_6–10_, diesel C_11–20_, and heavy oil C_21–40_) as a function of hexadecane conversion. Reaction conditions: *T* = 483 K, *P*_H_2_ or D_2__ = 30 bar, 1 g of hydrogenated or deuterated hexadecane, 25 mg of catalyst, 40 mL of *i*-C_5_, and 0.1–15 h of reaction.

In order to gain further insights into the elementary steps of polyolefin hydrogenolysis, we used linear model compounds with limited carbon chain length, i.e., *n*-hexadecane. The Ru/C-catalyzed hexadecane hydrogenolysis closely resembles PE hydrogenolysis. The linear correlation between hexadecane conversion with reaction time indicates zero-order kinetic for hydrogenolysis, attributed to strong hexadecane adsorption (Figure 4a). Hexadecane hydrogenolysis yielded C_1–15_ linear alkanes via stochastic C–C cleavage. At hexadecane conversion below 20%, the equivalent carbon fraction of C_6–15_ liquid products (Figure 4c,4d) was observed, but it was higher than the fraction of C_1–4_ gas products (Figure 4b). The carbon fraction in the products is uniformly distributed between liquid and gas products, implying a random C–C cleavage of central or internal C–C cleavage during hexadecane hydrogenolysis. With increasing hexadecane conversion, we observed a continuous shift of the product distribution toward shorter-chain hydrocarbons. This shift is attributed to secondary reactions of longer-chain alkanes, which reach a maximum in yield beyond 80% hexadecane conversion. The specific sequence of rates of secondary conversion (C_15_ > C_14_ > C_13_) shows that the adsorption strength also plays a major role during secondary C–C cleavage.

Moreover, we investigated the hydrogenolysis of squalane (2,6,10,15,19,23-hexamethyltetracosane) as an example of branched alkane hydrogenolysis. The consumption rate of squalane was found to be 0.15 mol/mol_Ru_/min below 20% conversion, which was an order of magnitude lower than that of hexadecane hydrogenolysis (2.7 mol/mol_Ru_/min). In analogy to the hydrogenolysis of *n*-C_5_ and *i*-C_5_, the lower reactivity is attributed to the significantly slower hydrogenolysis of C–C bonds involving tertiary C atoms (^3^C–^2^C and ^3^C–^1^C bonds) compared to ^2^C–^2^C or ^2^C–^1^C bonds.

When considering the primary C–C cleavage, the distribution of C_10_ products from squalane hydrogenolysis further demonstrates the C–C cleavage preference, which shows selectively dimetyloctane (Figure S8). Dimethyloctane is produced solely by ^2^C–^2^C cleavage, whereas the combination of ^3^C–^2^C and ^2^C–^2^C cleavages or ^3^C–^1^C cleavage can result in methylnonane formation. Negligible concentrations of *n*-decane, which requires secondary ^3^C–^1^C cleavage to form, and subsequent methane formation also reflect the preference for ^2^C–^2^C cleavage over ^3^C–^2^C or ^3^C–^1^C cleavage.

**Figure 5:**
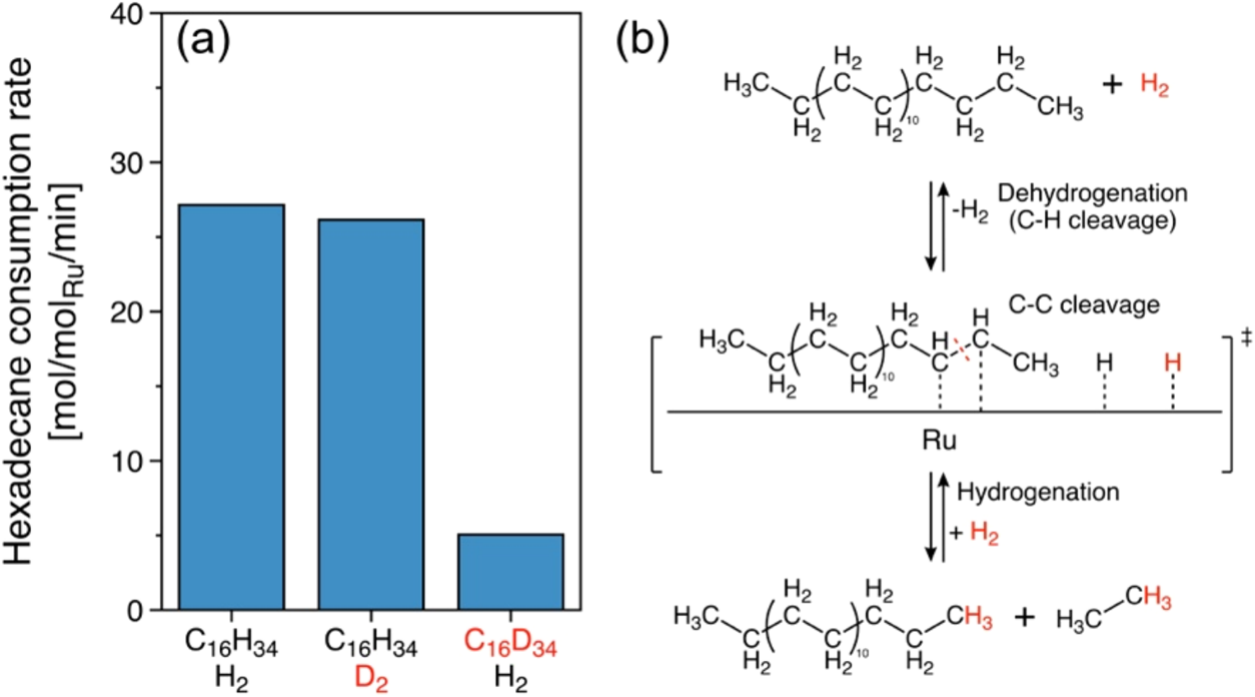
Dehydrogenation and hydrogenation of hexadecane upon hydrogenolysis. (a) Consumption rate of hydrogenated (C_16_H_34_) or deuterated hexadecane (C_16_D_34_) normalized to accessible surface Ru in H_2_ or D_2_. Reaction conditions: *T* = 483 K, *P*_H_2_ or D_2__ = 30 bar, 1 g of hydrogenated or deuterated hexadecane, 25 mg of catalyst, 40 mL of *i*-C_5_, and 0.1–15 h of reaction. The reaction rates are determined at conversion below 20%, ensuring a kinetic regime. (b) Proposed reaction pathway of Ru/C-catalyzed hexadecane hydrogenolysis with the assumption of *CH–CH* intermediate, including subsequent C–H (black) and central ^2^C–^2^C (red) cleavages, followed by hydrogenation (red).

The kinetic H/D isotope effects (KIEs) were investigated to probe the C–H cleavage and rehydrogenation after C–C cleavage products during hydrogenolysis (Figure 5a). The difference in the normalized consumption rate of n-hexadecane (C_16_H_34_) under H_2_ and D_2_ was 2.7 and 2.6 mol/mol_Ru_/min, respectively, i.e., no KIE between H_2_ and D_2_ (*k*_H_*/k*_D_ ∼ 1.1). This is attributed to comparable H_2_ and D_2_ dissociation as well as facile hydrogen addition by surface H and D from H_2_ and D_2_ after C–H and C–C cleavage on the Ru surface (Figure 5b). This further describe C-C cleavage can be kinetically relevant step upon hydrogenolysis than C–H cleavage and (re)hydrogenation. However, the hydrogenolysis rate of deuterated hexadecane (C_16_D_34_) in H_2_ was 5-fold reduced (*k*_C_16_H_34__*/k*_C_16_D_34__ ∼ 5) compared to *n*-C_16_H_34_ hydrogenolysis in H_2_. This suggests that the cleavage of C–H/C–D in hexadecane, but not rehydrogenation after C–C cleavage, accounts for hydrogenolysis kinetic.

**Figure 6:**
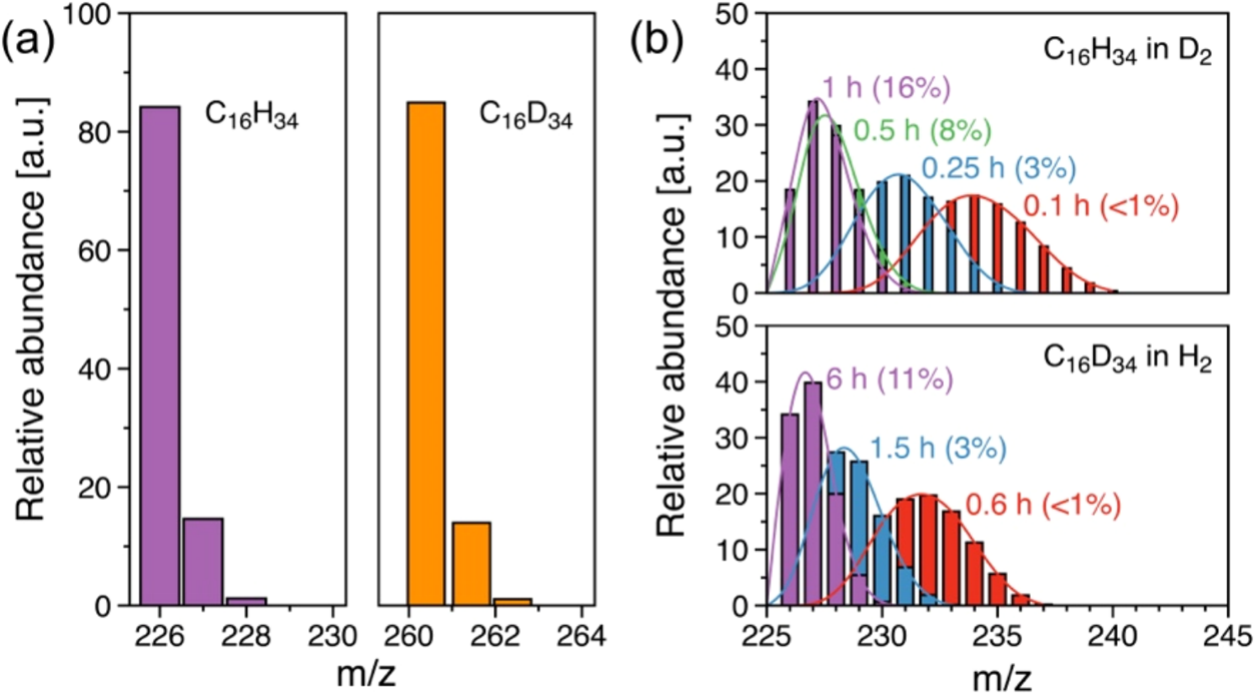
GC-MS mass spectra (a) hexadecane feedstock, i.e., C_16_H_34_ and C_16_D_34_, and (b) hexadecane in counterpart D_2_ (C_16_H_34_ in D_2_) or H_2_ (C_16_D_34_ in H_2_) as a function of reaction time with corresponding hexadecane conversion in the parentheses. Reaction conditions: *T* = 483 K, *P*_H_2_ or D_2__ = 30 bar, 1 g of hydrogenated or deuterated hexadecane, 25 mg of catalyst, 40 mL of *i*-C_5_, and 0.1–15 h of reaction. The reaction rates are determined at conversion below 20%, ensuring a kinetic regime.

Analyzing the mass spectra of unreacted hexadecane and products after hydrogenolysis allows us to estimate the degree of hydrogenation and dehydrogenation via the H–D exchange. For the parent C_16_H_34_ and C_16_D_34_ molecules, the primary mass fragments (before the hydrogenolysis) corresponded to the molecular weights of 226 and 260 g/mol, respectively, with the naturally abundant amount of ^13^C in the molecules (Figure 6a). The hexadecane conversion in D_2_ (C_16_H_34_–D_2_) at 0.1 h reaction time was <1% with no detectable gas and liquid products indicating minimal C–C cleavage through hydrogenolysis. The mass spectra of hexadecane showed a broad *m*/*z* distribution of 228–240 with the maximum abundance at 230 *m*/*z* (Figure 6b), i.e., 2–14 H–D exchanges per hexadecane molecule. This indicates that adsorption, desorption, and the reactions of dehydrogenation and hydrogenation can be regarded as quasi-equilibrated.

This finding allows us to quantify the H-D exchange rate of hexadecane with the deuteration rate as 3.2 × 10^2^ mmol_H-D_/mol_Ru_/s. On the other hand, for the counterpart C_16_D_34_ in H_2_, a comparable hexadecane conversion was observed at 0.6 h, where 23–33 D were exchanged with an 8-fold lower H-D exchange (40 mmol_H-D_/mol_Ru_/s). Taking into account the substantial hydrogenation rate, C–D cleavage in the dehydrogenation of C_16_D_34_ is concluded to lower the hydrogenolysis rate, probably because of the higher C–D dissociation energy of 341 kJ/mol in comparison to the C–H bond dissociation energy of 338 kJ/mol.^60^ Interestingly, with the extent of hexadecane conversion, the fraction of deuterium per hexadecane for both C_16_H_34_ in D_2_ and C_16_D_34_ in H_2_ was continuously reduced (Figure 6b) while the *m*/*z* = 72 of *i*-C_5_ solvent increased (Figure S9). This suggests that the deuterium of hexadecane was substituted by H from the solvent during hydrogenolysis. It is in agreement with the H-D exchange observed between deuterated polymer and hydrogen-containing solvents.^61^ Considering the negligible conversion of *i*-C_5_ during hexadecane hydrogenolysis, we conclude that the solvent in the presence of hexadecane (or other longer-chain alkanes) only undergoes C–H cleavage and H-D exchange by dehydrogenation and (re)hydrogenation but will experience minimal or no C–C cleavage.

**Figure 7:**
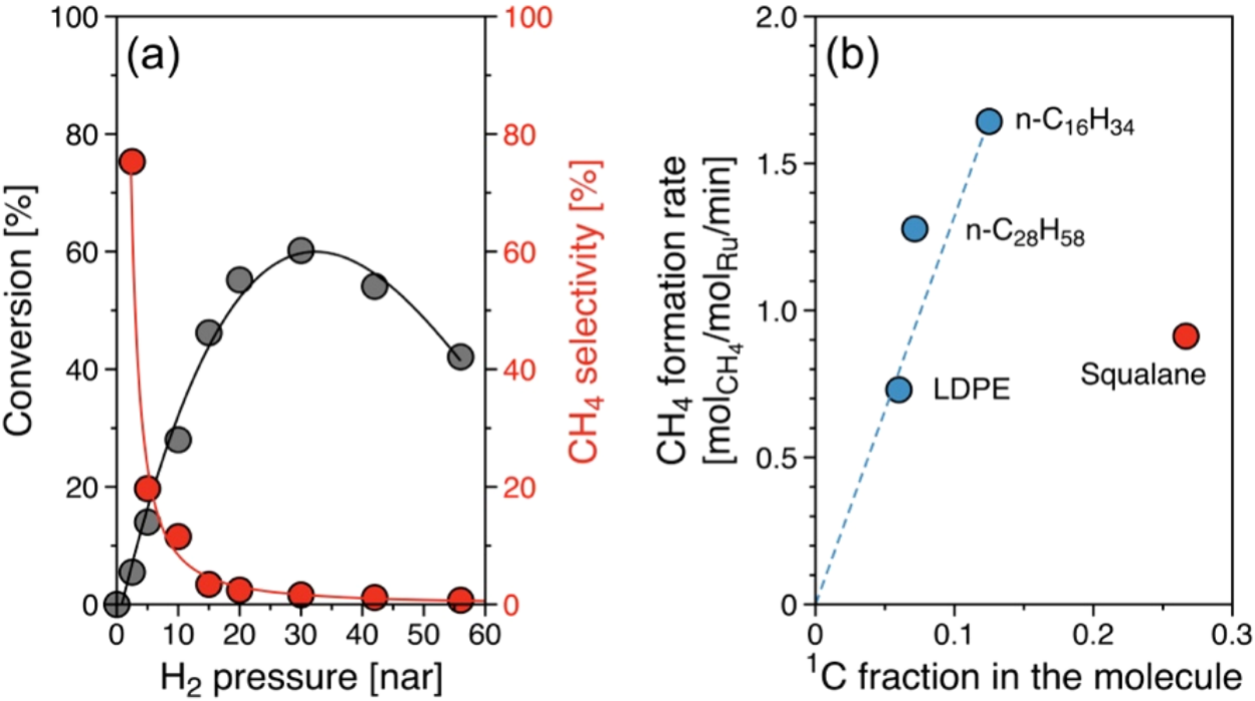
Methane formation upon Ru/C-catalyzed hydrogenolysis. (a) PE conversion and methane selectivity during PE hydrogenolysis as a function of hydrogen pressure. Reaction conditions: *T* = 483 K, *P*_H_2__ = 0–56 bar, 1 g PE, 150 mg of catalyst, 40 mL of iso-C_5_, and 2 h of reaction. (b) Methane formation rate during PE as well as linear (*n*-C_16_H_34_ and *n*-C_28_H_58_) and branched (squalane, C_30_H_62_) hydrogenolysis, as a function of primary carbon fraction in the molecules. Reaction conditions: *T* = 483 K, *P*_H_2__ = 30 bar, 1 g of PE or hydrocarbon, 25 mg of catalyst, 40 mL of iso-C_5_, and 2–5 h of reaction. The reaction rates are determined at comparable conversion (∼20%).

Expectedly, the H_2_ partial pressure significantly impacted the alkane hydrogenolysis rates. Figure 7 shows PE conversion and methane selectivity as functions of hydrogen pressure. PE conversion was negligible in the absence of H_2_ (Figure 7a) and greatly increased as H_2_ pressure increased from 2.5 to 30 bar. However, the conversion decreased as H_2_ pressure exceeded 30 bar. The decrease in PE conversion is attributed to high hydrogen coverage of the surface, with hydrogen competing for adsorption sites with PE. This finding is also in good agreement with the negative reaction order of H_2_ for alkane hydrogenolysis.^44,55,59,62^

From a mechanistic point of view, hydrogenolysis proceeds through the following elementary reactions: (i) stepwise (full) dehydrogenation forming a strong C-metal bond on the surface, (ii) C–C bond cleavage, and (iii) (re)hydrogenation followed by desorption.^44,62−67^ To bind dissociated hydrogen upon C–H bond cleavage, free adjacent metal sites are beneficial (the surface-bound hydrogen is likely mobile on the surface^68−71^). In the absence of H_2_, the dehydrogenation of the adsorbed alkane leads to the formation of unreactive surface intermediates and desorption of H_2_. Thus, initially, the presence of H_2_ increases the reaction rate by facilitating the rehydrogenation of the reacted fragments of the first two elementary reaction sequences. Increasing H_2_ pressure, however, leads to the increase of H* coverage on the surface and ultimately lowers the PE conversion. Interestingly, methane selectivity was substantially reduced with increasing H_2_ pressure, reflecting the lower requirement of available binding sites for dissociated C–H groups for ^2^C–^2^C cleavage, consequently suppressing consecutive terminal C–C cleavage.

Methane selectivity was relatively invariable along the PE conversion degree (Figure 7b). At a PE conversion of 45% the methane formation rate was reduced by 4-fold from 0.53 to 0.13 mol_CH_4__/mol_Ru_/min as the hydrogen pressure was increased from 15 to 56 bar. Thus, we conclude that higher H_2_ pressure leads to preferential hydrogenolysis of central C–C bonds because these require a lower number of available binding sites for H*.

Exploring the methane formation further using linear and branched model compounds, Figure 7b compares methane formation rate during hydrogenolysis of PE, *n*-hexadecane (*n*-C_16_H_34_), octacosane (*n*-C_28_H_56_), and squalane as a function of primary carbon fraction. The fraction of primary (^1^C), secondary (^2^C), and tertiary (^3^C) carbon atoms in PE was determined by solid-state NMR as shown in Figure S10. The methane formation rate at comparable conversion (∼20%) showed a linear correlation with the primary carbon fraction, i.e., the concentration of terminal carbon in the reacting substrate. Even though a higher fraction of primary carbon is present in squalane (0.27) than in the other alkanes studied, the methane formation rate of 0.91 mol_CH_4__/mol_Ru_/min was lower than for the linear hydrocarbons, hexadecane (1.64 mol_CH_4__/mol_Ru_/min) and octacosane (1.28 mol_CH_4__/mol_Ru_/min). This is consistent with the previously noted lower reactivity of ^1^C–^3^C bonds that dominate in squalane.

## 3 Conclusions

Ru offers a path to deconstructing polyolefins via hydrogenolysis under supercritical conditions while minimizing methane formation. Ru-catalyzed hydrogenolysis of polyethylene and alkanes, such as hexadecane and squalane, in the presence of *i*-C_5_ as a solvent occurs with satisfying rates above supercritical conditions of 463 K. Hydrogenolysis of alkanes with intermediate chain length as model compounds shows that C–C bond cleavage occurs stochastically, however, with a preference for cleaving C–C bonds between primary and secondary carbon atoms over those involving tertiary carbon atoms. Therefore, a light-branched hydrocarbon (iso-butane is predicted to be ideal) as solvent allows for the selective C–C cleavage of polyethylene and hexadecane, minimizing solvent conversion. H-D exchange and the kinetic isotope effect upon hydrogenolysis of deuterated hexadecane in H_2_ and *n*-hexadecane in D_2_ led us to conclude that the reaction starts with extensive C–H cleavage (local dehydrogenation), followed by C–C cleavage of the dehydrogenated carbon atoms and hydrogenation of the two surface fragments. We conclude that C–C cleavage is the kinetically rate-determining step, where C–D cleavage can lower the hydrogenolysis rate due to higher dissociation energy than C–H cleavage by 3 kJ/mol. The supercritical state of the solvents (463–483 K) changes the binding of adsorbed H_2_ on the catalyst surface, leading to a more facile dehydrogenation of the CH_*x*_ bonds involved in the C–C scission. Increasing the H_2_ chemical potential and diluting the longer-chain hydrocarbon minimizes methane formation, as hydrogenolysis at terminal C–C bonds requires a larger number of available surface sites to break the C–C bond. These findings now elucidate a full mechanistic understanding of the stochastic C–C cleavage in polyolefin hydrogenolysis particularly under supercritical conditions. It also leads to a significant reduction of methane production and advancing the development of new technologies for converting plastic waste into valuable chemicals.

## 4 Experimental Methods

### 4.1 Chemicals

Polyethylene (PE) was purchased from Sigma-Aldrich as powder, with a *M*_w_ and *M_n_* of ∼4000 g/mol–1700, respectively. Activated carbon-supported Ru, Rh, Pd, Ir, and Ni catalysts at 5 wt % were purchased from Sigma-Aldrich. 2-Methylbutane, *n*-butane, *n*-hexane, *n*-butylcyclohexane, and ethyl acetate for solvent and external standard were also purchased from Sigma-Aldrich.

### 4.2 Characterization

#### 4.2.1 Elemental Analysis

The metal contents in the catalysts were determined using a PerkinElmer Optima 7300DV ICP-OES instrument equipped with a cyclonic spray chamber and a Meinhard nebulizer. Prior to analysis, the samples (50–60 mg) were digested with HNO_3_ (3 mL)/HCl (2 mL)/HF (0.5 mL)/H_2_O (0.5 mL) in a sealed vessel at 483 K for 30 min. After cooling to room temperature, 1.5 mL of saturated boric acid solution was added and then heated at 453 K for 20 min.

#### 4.2.2 Nitrogen Physisorption

The porosity was measured by N_2_-physisorption at 77 K using Micrometrics ASAP 2020. Prior to N_2_ adsorption, each sample was degassed at 573 K for 5 h under vacuum (10^–3^ mbar). The Brunauer–Emmett–Teller, Barrett–Joyner–Halenda, and t-plot methods were employed to determine the specific surface area, mesopore volume, and micropore volume, respectively.

#### 4.2.3 Solid-State ^1^H and ^13^C Magic-Angle Spinning (MAS) NMR

Solid-state MAS NMR measurements were carried out using a Varian Inova wide-bore 300 MHz NMR spectrometer equipped with a 7.5 mm commercial Vespel MAS NMR probe and a commercial heating stack for variable temperature experiments. The corresponding Larmor frequencies for ^1^H and ^13^C were 299.97 and 75.43 MHz, respectively. The samples were stored in a nitrogen-filled glovebox, in which a MAS NMR rotor with 300 μL volume was packed.^72−74^ For single-pulse ^1^H and ^13^C MAS NMR experiments, the MAS frequency was 4 kHz, and 128–2000 spectra were acquired at 293–423 K depending on ^1^H or ^13^C MAS NMR experiment with a 45° angle pulse, 2 μs of pulse width, and 20 s of recycle delay of 20 s. All of the spectra were referenced to TMS (0 ppm) by using adamantane as a second reference (38.48 ppm for its downfield ^13^C peak and 1.82 ppm for the center band of ^1^H).

#### 4.2.4 Hydrogen Chemisorption

H_2_ chemisorption was conducted with Micromeritics ASAP 2020 in chemisorption mode. The catalyst was outgassed under vacuum (10^–3^ mbar) at room temperature, followed by reduction under H_2_ (50 mL/min) at 623 K. The first adsorption isotherm was recorded at room temperature from 0.1 mbar to 600 mbar at 323 K. After the evacuation at 323 K under vacuum (10^–3^ mbar) for 1 h, the second isotherm was measured. The chemisorbed H_2_ was then quantified by the difference between the first and second isotherms, which was then extrapolated to zero H_2_ pressure. The stoichiometry factor between dissociated H_2_ and the active metal was assumed to be 1.0 (H/Ru).^75−77^

### 4.3 Catalytic Hydrogenolysis

Catalytic hydrogenolysis of PE in solvents and of the neat solvent was performed at 443–503 K using a 100 mL Hastelloy PARR reactor. In a typical reaction, 40 mL of solvent, 1–3 g of PE, and 20–150 mg of catalyst were loaded into the autoclave reactor. The reactor was sealed, and the air was removed by pressurizing with H_2_ and venting it at least five successive cycles. On the final cycle, the reactor was pressurized with H_2_ to the desired pressure at 298 K and then heated to reaction temperature (443–503 K) under vigorous stirring at 700 rpm. After the reaction, the reactor was immediately quenched to a temperature below 293 K with an ice/water mixture. The gas products in the headspace were transferred directly to a 5 L gas sampling bag (Tedlar) and analyzed by gas chromatography with thermal conductivity detection (GC-TCD) (Inficon Micro GC Fusion gas analyzer with a four-module chassis). Response factors (RF) for each gas product were determined from calibration curves relating the area to the carbon mole fraction, which was further used for quantifying the carbon yield of the gas products with the known headspace volume and pressure in the reactor:
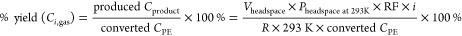
where *R* is the gas constant.

When the pressure vessel was vented entirely, it was disassembled, and the remaining liquid and solids were transferred together via pipet to a preweighed 40 mL glass vial. The liquids were separated from the solids by filtration and analyzed by gas chromatography-flame ionization detection (GC-FID) (Agilent 7890A GC, DB-5 column, Agilent 7693 autosampler), where the liquid products were quantified with external standard of *n*-butylcyclohexane. To determine the mass of reacted PE, all remaining solids were collected, dried overnight at 353 K, and weighed. The amount of catalyst was subtracted from the final weight. The carbon balance was calculated by the comparison between the sum of carbon moles in gas and liquid products and the converted carbon moles in the PE, whereas the conversion of solvent was disregarded due to negligible reactivity of solvent hydrogenolysis and the absence of products from solvent hydrogenolysis after reaction, such as *i*-C_4_ from *i*-C_5_ solvent. The PE carbon fraction was estimated using ^1^H NMR (Figure S10a), resulting in the molar percentage of primary (6 mol %, −CH_3_) and secondary (94 mol %, −CH_2_−) carbon atoms, whereas the fraction of tertiary carbons in PE was negligible, corresponding to 0.85 mol %. Therefore, the equation for carbon balance can be expressed as
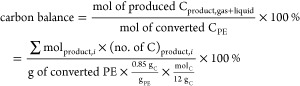
The carbon balance for the selected experiments has been listed in Table S2, showing reliable values (>95%) through 93–99% PE conversion at different temperatures and reaction times.

## Supporting Information Available

Physicochemical properties of Ru/C catalyst; phase envelop diagram for the solvents; product distribution of PE hydrogenolysis; and solid-state ^1^H and ^13^C magic-angle NMR of PE at 373 K (PDF)
