# Supplementary material for: Ru-Catalyzed Polyethylene Hydrogenolysis under Quasi-Supercritical Conditions
Source: JACS Au. 2025 Apr 16;5(4):1760–70. doi: 10.1021/jacsau.5c00006 (PMC12042042; doi:10.1021/jacsau.5c00006)
Supplement: Supplementary file 1 — au5c00006_si_001.pdf [file au5c00006_si_001.pdf]

Supporting information

Ru-catalyzed polyethylene hydrogenolysis under quasi-supercritical conditions

Sungmin Kim,[a,#] Boda Yang,[a,#] Oliver Y. Gutiérrez,[a] Wei Zhang,[a] Carlos Lizandara-Pueyo,[b] Piyush Ingale,[c] Ivana Jevtovikj,[c] Reni Grauke,[c] Janos Szanyi,[a] Huamin Wang,[a] Stephan A. Schunk,[b,c,d] Johannes A. Lercher[a,e,*]

[a]Institute for Integrated Catalysis and Physical Science Division,
Pacific Northwest National Laboratory, Richland, Washington 99354, United States
[b] BASF SE, Carl-Bosch-Straße 38, 67056 Ludwigshafen am Rhein, Germany
[c] hte GmbH, Kurpfalzring 104, 69123 Heidelberg, Germany
[d]Universität Leipzig, Institut für Technische Chemie, Linnéstraße 3, 04103 Leipzig, Germany
[e]Department of Chemistry and Catalysis Research Institute,
TU München, Lichtenbergstrasse 4, 85748 Garching, Germany

# The authors equally contributed

Corresponding authors: Johannes.Lercher@pnnl.gov (J. A. Lercher)

Table S1. Summary of physicochemical properties of Ru/C

Catalyst	Ru[a] [wt.%]	S_{BET}[b] [m^2/g]	V_{pore}[b] [cm^3/g]	H_2 uptake [μmol$_{H2}$/g]	Surface Ru[c] [μmol$_{Ru}$/g]	Ru dispersion [%]
Ru/C	4.92	590	0.43	62	124	25.5
Ru/SiO_2	4.82	130	0.21	32	64	13.6
Ru/Al_2O_3	5.01	88	0.17	34	68	13.8
Ru/SiO_2-Al_2O_3	4.75	167	0.31	31	62	13.2

[a]The weight percentage of Ru was determined by ICP-OES. [b]The specific surface area, pore volume, and pore radius were calculated using BET and BJH models. [c]The quantity of surface Ru in the reduced materials was determined by H_2 chemisorption using a stoichiometry factor of 1.0 of H/Ru.

Table S2. Carbon balance based on the gas and liquid products by comparison to converted PE

Catalyst	H_2 pressure [bar]	Temperature [°C]	Reaction time [h]	PE Conversion [%]	Carbon balance[a] [%]
Rh/C	30	210	2	16	98
Ru/C[b]		190	2	19	96
Ru/C[b]		210	2	15	95
Ru/C[b]		230	2	17	93
Ru/SiO_2[c]		210	2	48	99
Ru/Al_2O_3[c]		210	2	55	95
Ru/SiO_2-Al_2O_3[c]		210	2	42	98

[a]The carbon balance was calculated by comparison between the sum of carbon moles in gas and liquid products and the converted carbon moles in the PE. The PE carbon fraction was estimated using ^{1}H NMR (Figure S10a), resulting in the molar fraction of primary (6 mol%, -CH_3) and secondary (94 mol%, -CH_2-) carbons, whereas negligible tertiary carbon fraction in PE, corresponding 0.85 of carbon mass fraction. [b]The PE conversion were measured at kinetic regime, i.e., below 20% of PE conversion. The reaction condition: T = 463–503 K, P_{H2} = 30 bar, 1 g of PE, 20-300 mg of catalyst, 40 mL of i-C5, and 0.5-2 h of reaction. [c]The carbon balance is represented for Figure S1b.

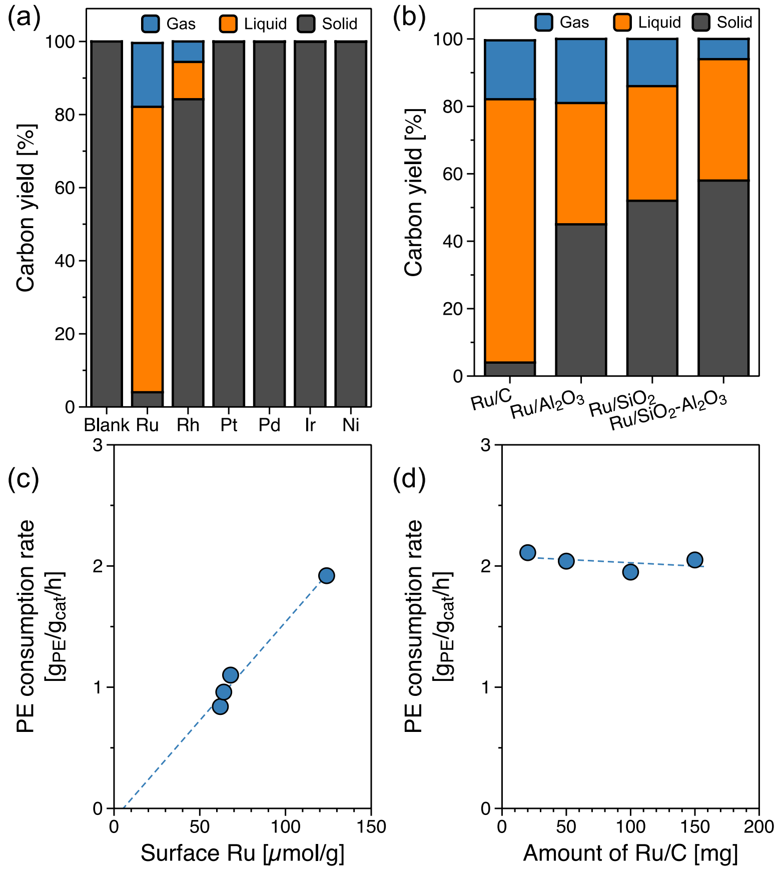

Figure S1. Carbon yield in gas (C_{1-4}), liquid (C_{5-40}), and solid residue after PE hydrogenolysis on (a) carbon-supported transition metal catalysts and (b) Ru catalysts supported on carbon or metal oxide. The carbon balance was listed in Table S2. (c) PE consumption rate as a function of surface Ru quantified by hydrogen chemisorption of carbon or metal oxide supported Ru, where surface Ru (Table S1). Reaction conditions: T = 483 K, P_{H2} = 30 bar, 1 g of PE, 150 mg of catalyst, 40 mL of i-C_5, and 2 h of reaction. (d) PE consumption rate as a function of the amount of Ru/C catalyst, where the PE conversion was measured in the kinetic regime below 20% PE conversion. Reaction conditions: T = 483 K, P_{H2} = 30 bar, 1 g of PE, 20–150 mg of catalyst, 40 mL of i-C_5, and 0.3–2 h of reaction.

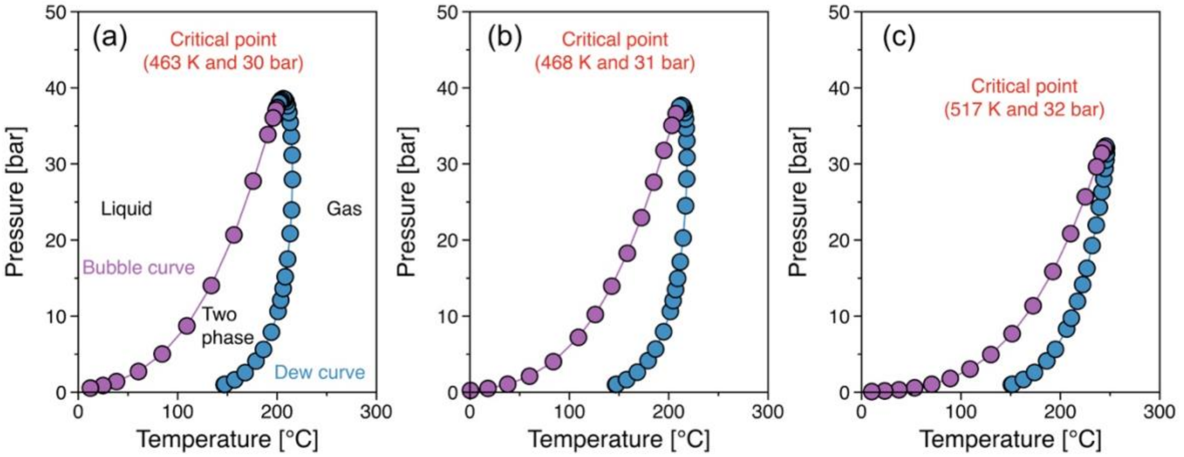

Figure S2. Pressure-temperature projection of phase diagram (phase envelop) for the mixture of hexadecane and solvent: (a) isopentane, (b) n-pentane, and (c) n-hexane. Aspen plus with Soave-Redlich-Kwong equation of state estimates phase envelop, which allows to predict critical points for the mixture.

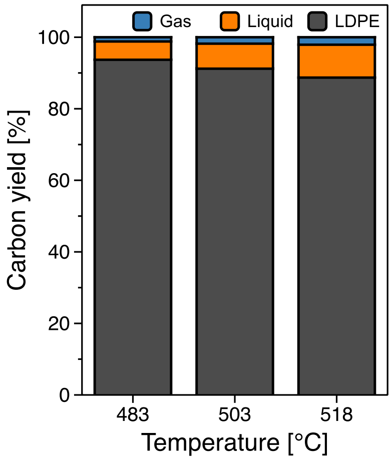

Figure S3. Carbon yield in gas (C_{1-4}), liquid (C_{5-40}), and solid residue after Ru/C-catalyzed PE hydrogenolysis with n-C_6. The reaction conditions: T = 483–518 K, P_{H2} = 30 bar, 1 g of PE, 30 mg of catalyst, 40 mL of n-C_6, and 2 h of reaction.

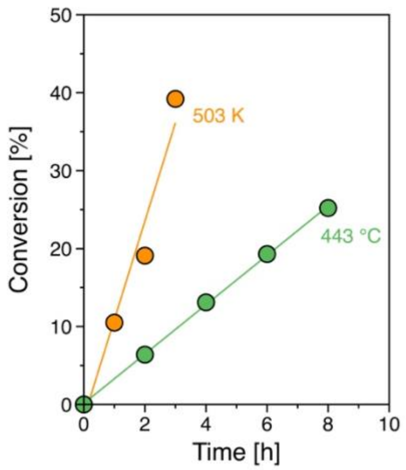

Figure S4. PE conversion with reaction time at 443 and 503 K. The reaction conditions: P_{H2} = 30 bar, 1 g of PE, 20 (503 K) and 300 (443 K) mg of catalyst, 40 mL of i-C_5, and 0.1–10 h of reaction.

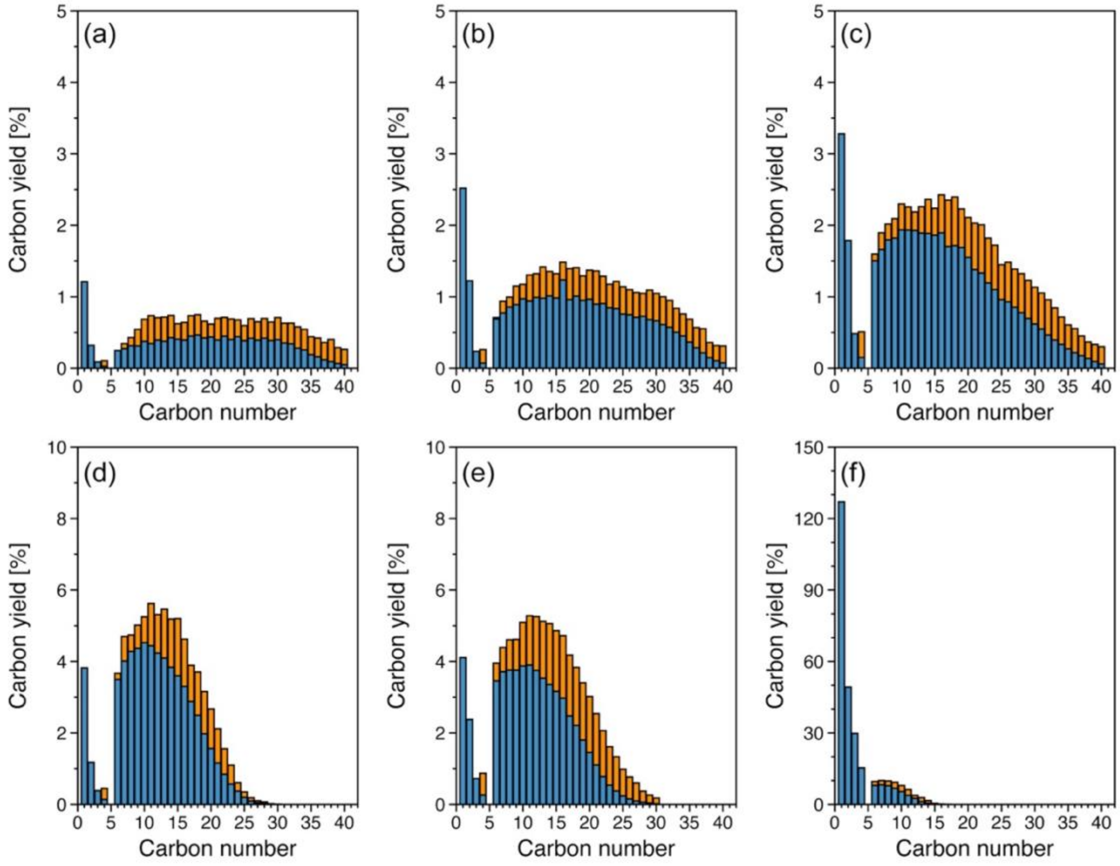

Figure S5. Carbon yield of gas and liquid products at different PE conversion upon hydrogenolysis: (a) 26%, (b) 50 %, (c) 73%, (d) 86%, (e) 96, and (f) excess hydrogenolysis. The reaction conditions: T = 483 K, P_{H2} = 30 bar, 1 g of PE, 150 mg of catalyst, 40 mL of i-C_5, and 0.1–20 h of reaction. The blue and orange bars in the figure represented linear and branched alkanes, respectively.

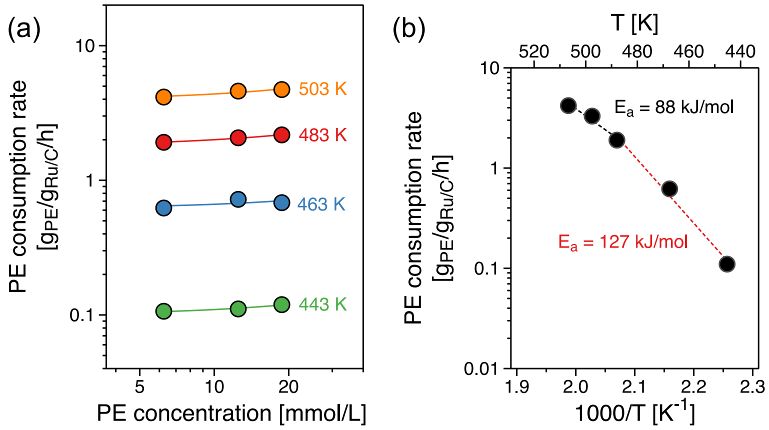

Figure S6. PE consumption rate at 443–503 K as a function of (a) PE concentration (1-3 g of PE in 40 mL isopentane) in isopentane solvent, and (b) temperature with 1 g PE in 40 mL isopentane. The reaction condition: T = 443–503 K, P_{H2} = 30 bar, 1–3 g of PE, 20-300 mg of catalyst, 40 mL of i-C_5, and 2 h of reaction. The solid lines are fits to the Arrhenius equation. The reaction rates are determined at conversion below 20%, ensuring kinetic regime.

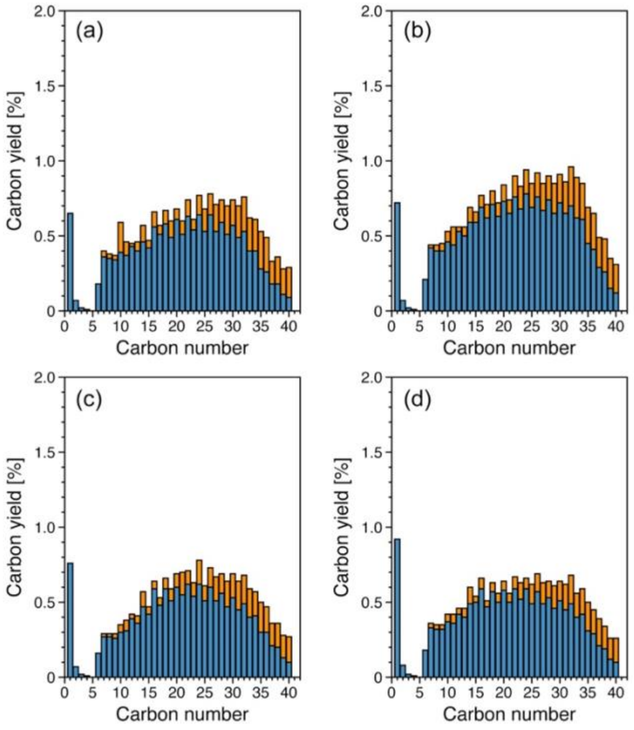

Figure S7. Carbon yield of gas and liquid products from PE hydrogenolysis under the kinetic regime (≈ 20% PE conversion) at different temperatures varying the amount of catalyst and reaction time: (a) 200 mg at 443 K for 10 h, (b) 100 mg at 463 K for 8 h, (c) 25 mg at 483 K for 4 h and (d) 25 mg at 503 K for 2 h. The reaction conditions: P_{H2} = 30 bar, 1 g of PE, 200 mg, 40 mL of i-C_5. The blue and orange bars in the figure represented linear and branched alkanes, respectively.

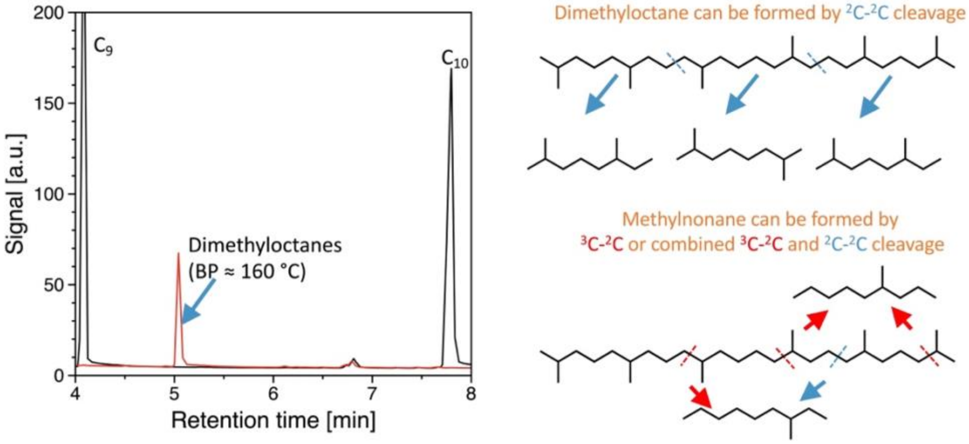

Figure S8. GC-FID chromatogram of C_{10} products after squalane hydrogenolysis. The reaction conditions: T = 483 K, P_{H2} = 30 bar, 1 g of squalane, 150 mg of catalyst, 40 mL of i-C_5, and 2 h of reaction. The scheme next to the chromatogram describes ^{3}C-^{2}C (red) and ^{2}C-^{2}C (red) cleavage producing dimethyloctane and methylnonane from squalane hydrogenolysis.

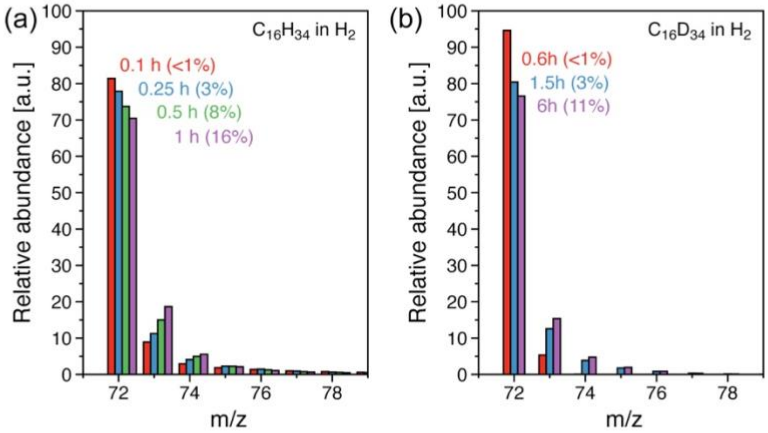

Figure S9. GC-MS mass spectra of i-C_5 solvent during hexadecane hydrogenolysis with (a) $C_{16}H_{34}$ in D_2 and (b) $C_{16}D_{34}$ in H_2 as a function of reaction time with corresponding hexadecane conversion in the parentheses. The reaction conditions: T = 483 K, $P_{H2\ or\ D2}$= 30 bar, 1 g of hydrogenated or deuterated hexadecane, 25 mg of catalyst, 40 mL of i-C_5, and 0.1–15 h of reaction.

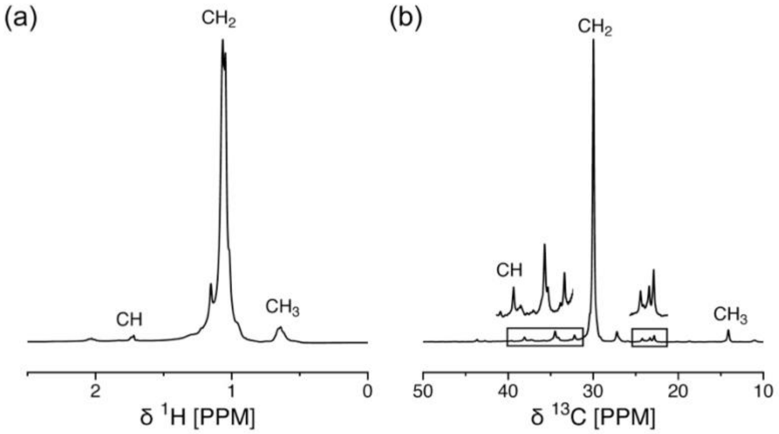

Figure S10. Solid-state (a) ^{1}H and (b) ^{13}C Magic-angle NMR spectra of PE at 373 K. The primary (CH_3), secondary (CH_2), and tertiary (CH) carbon are assigned, while the inset in Figure S10b represents CH_2 in the branched chains on PE.[1, 2]

References

1. Jung, M.; Lee, Y.; Kwak, S.; Park, H.; Kim, B.; Kim, S.; Lee, K. H.; Cho, H. S.; Hwang, K. Y., Analysis of Chain Branch of Polyolefins by a New Proton NMR Approach. Anal. Chem. 2016, 88 (3), 1516-1520.
2. Eselem Bungu, P. S.; Pasch, H., Comprehensive analysis of branched polyethylene: the multiple preparative fractionation concept. Polym. Chem. 2017, 8 (31), 4565-4575.
